# A Rare Presentation of Cholangitis Associated With Invasive Streptococcus Pyogenes

**DOI:** 10.7759/cureus.62146

**Published:** 2024-06-11

**Authors:** Dominique M Ebedes, Jennifer Caputo-Seidler

**Affiliations:** 1 Internal Medicine, University of South Florida Morsani College of Medicine, Tampa, USA

**Keywords:** endoscopy, pancreatic cancer, sepsis, cholangitis, streptococcus pyogenes

## Abstract

*Streptococcus pyogenes* (*S. pyogenes*) is a gram-positive, facultative anaerobic bacterium that appears as cocci in chains and commonly causes skin infections and pharyngitis. Here, we present a very uncommon case of cholangitis associated with invasive *S. pyogenes* infection in a 34-year-old man with metastatic pancreatic adenocarcinoma who presented with fever, right upper quadrant pain, jaundice, altered mental status, and hypotension. The patient underwent a percutaneous transhepatic cholangiogram, showing moderate dilatation of intrahepatic biliary ducts with obstruction of the proximal common bile duct, and an internal/external biliary drain was placed to allow for the flow of bile. Blood cultures grew *S. pyogenes*. Biliary fluid culture obtained at the time of drain placement grew *S. pyogenes*, *lactobacilli*, and *saccharomyces*. The patient was treated with ampicillin-sulbactam and fluconazole. While the patient recovered from his sepsis, he died within weeks of diagnosis due to complications of metastatic disease.

## Introduction

*Streptococcus pyogenes* (*S. pyogenes*) is a gram-positive, facultative anaerobic bacterium that appears as cocci in chains and constitutes a major human-specific bacterial pathogen. Manifestations of *S. pyogenes* infection range from relatively mild local infections, such as skin infections (impetigo, erysipelas, cellulitis, necrotizing fasciitis) and pharyngitis (strep throat), to more systemic life-threatening conditions like toxic shock syndrome and necrotizing fasciitis [1]. While the precise epidemiological patterns of invasive group A streptococcus infection are yet to be clearly delineated, existing data suggest that there has been a rise in these infections in developed countries [2]. The increase in incidence of *S. pyogenes* is theorized to result from the expansion of particularly virulent clones [2]. In this report, we present a rare case of invasive *S. pyogenes* infection associated with cholangitis.

Cholangitis is a life-threatening infection of the biliary tree typically caused by coliform enteric bacteria such as *Escherichia coli* (*E. coli*) (25%-50%), *Klebsiella* (15%-20%), *Enterococcus* (10%-20%), and *Enterobacter* species (5%-10%). Anaerobic bacteria, such as *Bacteroides fragilis* and *Clostridium perfringens*, can also less commonly cause cholangitis, particularly in patients with previous biliary surgery and in the elderly [3]. To date, we have only identified one other report of invasive *S. pyogenes* infection associated with cholangitis in the literature [4].

## Case presentation

A 34-year-old man with a newly diagnosed pancreatic adenocarcinoma with metastases to the liver presented to the emergency department with nausea and vomiting. He was scheduled to initiate treatment with FOLFIRINOX (leucovorin calcium (folinic acid), fluorouracil, irinotecan hydrochloride, and oxaliplatin) the week his symptoms began but received no chemotherapy or immunosuppression prior to this.

**Figure 1:**
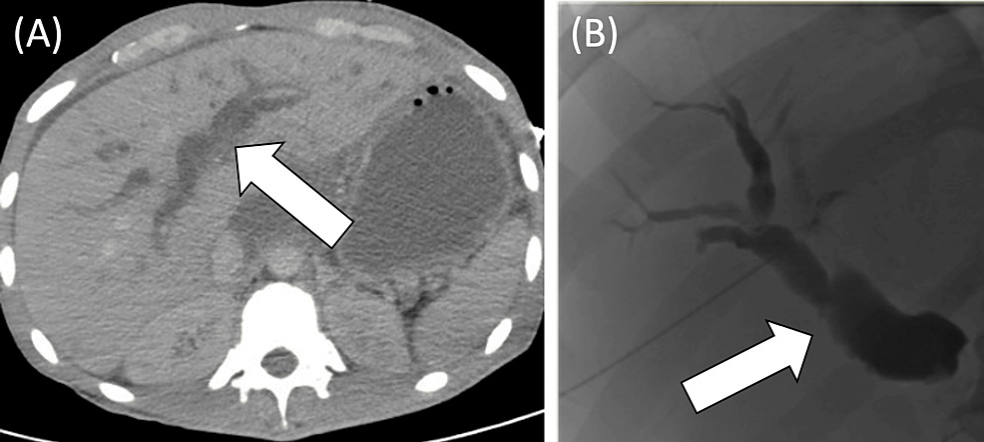
Axial CT scan (A) and cholangiogram (B) demonstrate biliary dilation consistent with cholangitis The white arrows indicate dilated bile ducts. The CT scan (A) was obtained on day 1 when the patient presented to the emergency department, and the cholangiogram (B) was obtained on day 2. Image credits - Dominique Ebedes

On presentation, the patient was febrile (101.8 F), tachycardic (160 bpm), and hypotensive (83/38) with altered mental status, jaundice, and right upper quadrant pain on physical exam. Labs revealed a normal white blood cell count (WBC of 9). Total bilirubin was elevated to 14 mg/dL from the patient’s baseline of 6 mg/dL (normal level < 1.3 mg/dL). A CT abdomen was performed, revealing an interval increase in intra and extrahepatic biliary ductal dilatation (Figure 1).

The patient was admitted to the medical intensive care unit with a probable diagnosis of septic shock secondary to a biliary source. A day after the patient was admitted, his blood cultures returned positive for *group A streptococcus*. The interventional radiology team was consulted, and the patient underwent a percutaneous transhepatic cholangiogram. This revealed moderate dilatation of intrahepatic biliary ducts with obstruction of the proximal common bile duct (Figure 1). An internal/external biliary drain was placed to allow for the flow of bile.

By this time, blood cultures had speciated with *S. pyogenes*. The patient’s clinical presentation remained most consistent with acute cholangitis. A thorough physical examination revealed no evidence of skin or soft tissue infection, no evidence of port site infection, and no pharyngitis. Biliary fluid culture obtained at the time of drain placement grew *S. pyogenes*, *lactobacilli*, and *saccharomyces*. This finding, in particular, suggested cholangitis as the source of the patient’s *S. pyogenes* bacteremia, and notably was the first instance we could find in the literature in which *S. pyogenes* was isolated from a biliary fluid culture. Amplicillin-sulbactam was prescribed to cover *S. pyogenes* and *lactobacillus*, and oral fluconazole was prescribed for *saccharomyces*. Over the next week, the patient's infectious symptoms resolved, however, he passed shortly after due to metastatic disease.

## Discussion

In this report, we presented a case of cholangitis caused by *S. pyogenes* in a septic 34-year-old male with recently diagnosed pancreatic adenocarcinoma. This is the first case report in which *S. pyogenes* was isolated from a biliary fluid culture.

The other case in the English literature occurred in a 52-year-old man with a diagnosis of pancreatic cancer, who underwent cholecystojejunostomy and gastrojejunostomy for pancreatic cancer complicated by obstructive jaundice and *E. coli* bacteremia for which he underwent percutaneous transhepatic cholangiography with biliary drainage and percutaneous internal biliary stent placement [3]. Two months later, he was admitted with chills, abdominal pain, jaundice, and elevated serum bilirubin, alkaline phosphatase, and gamma-glutamyl transpeptidase. Unlike our patient, this patient remained afebrile. Like our patient, he had no evidence of pharyngeal erythema, lung, or skin infection, and his white blood cell count was within normal limits. CT abdomen revealed intra and extrahepatic biliary ductal dilatation. Blood cultures grew *S. pyogenes*. The patient was treated with ampicillin and endoscopic biliary stent placement, leading to marked clinical improvement. While this case made a strong argument for the diagnosis of *S. pyogenes* cholangitis (elevated biliary labs, jaundice, biliary dilation on CT, positive *S. pyogenes* blood culture, and responsiveness to ampicillin and biliary drainage), the study was limited in that the pathogen was not isolated from a bile sample. In our case, biliary fluid culture was obtained at the time of drain placement and subsequently also grew *S. pyogenes*. This more definitively suggested cholangitis as the source of the patient’s bacteremia.

**Figure 2:**
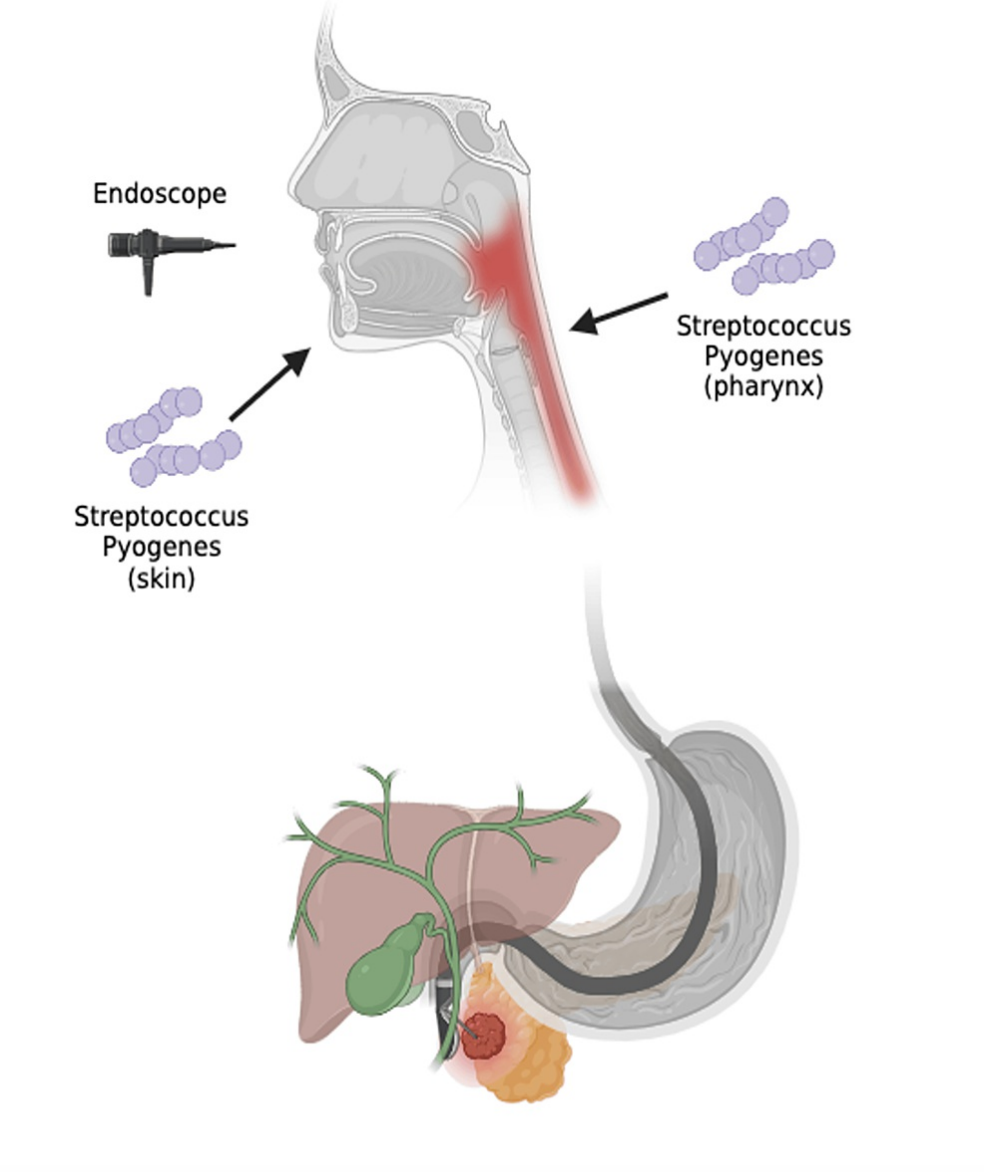
Proposed mechanism by which S. pyogenes is introduced into the biliary system during endoscopy. Image created using BioRender.com; Image credits - Dominique Ebedes

We hypothesize that the most likely factor implicated in both our patient’s infection and the previously reported case is their history of endoscopy. Endoscopy is an important known risk factor for the development of cholangitis [5]. During an endoscopic procedure, the endoscope traverses both the skin near the oral cavity and the pharynx (a more typical site for *S. pyogenes* infections) before arriving at the digestive system. We theorize that this may allow for the introduction of *S. pyogenes* from the skin or pharynx into the biliary system, resulting in cholangitis (Figure 2). Of course, while this mechanism is compelling, it can by no means be proven by this singular case. Other possible compelling mechanisms of pathogenic spread include bacteremia seeding the biliary tree and the drain placement procedure seeding infection from the throat. 

## Conclusions

While cholangitis is typically caused by gram-negative enteric bacteria, such as *E. coli* and *Klebsiella*, other non-enteric organisms, such as *S. pyogenes,* are a rare cause of this infection. We provide the second case of *S. pyogenes-*associated cholangitis and the first case in which *S. pyogenes* was isolated from a biliary fluid culture. Endoscopy is an important known risk factor for the development of cholangitis. Although less common, *S. pyogenes* appears to be a potential etiology for post-endoscopy cholangitis. It is important that physicians maintain a high suspicion for this diagnosis in patients presenting with sepsis, altered mental status, jaundice, and right upper quadrant pain after endoscopy, as cholangitis can progress rapidly and cause mortality if it is not identified and treated in a timely fashion. More research and reporting are needed to identify the true prevalence of post-endoscopic retrograde cholangiopancreatography (ERCP)​​​​​​ *S. pyogenes* cholangitis. Patients with pancreatic cancer who undergo repeated ERCP procedures may be at particular risk.
